# Menopausal status induces vaginal dysbiosis in women with human papillomavirus infection

**DOI:** 10.1038/s41598-024-56314-9

**Published:** 2024-03-26

**Authors:** Kyeong A So, Soo Young Hur, Chi Heum Cho, Jae Kwan Lee, Seok Ju Seong, Dae Hoon Jeong, Moran Ki, Tae Jin Kim

**Affiliations:** 1 Department of Obstetrics and Gynecology, Konkuk University School of Medicine, 120-1, Neungdong-ro, Gwangjin-gu, Seoul, 05030 Republic of Korea; 2 Department of Obstetrics and Gynecology, Seoul St. Mary’s Hospital, The Catholic University, Seoul, Republic of Korea; 3 Department of Obstetrics and Gynecology, Keimyung University Dongsan Medical Center, Daegu, Republic of Korea; 4 Department of Obstetrics and Gynecology, College of Medicine, Korea University Guro Hospital, Korea University, Seoul, Republic of Korea; 5 Department of Obstetrics and Gynecology, CHA Gangnam Medical Center, CHA University College of Medicine, Seoul, Republic of Korea; 6 Department of Obstetrics and Gynecology, Busan Paik Hospital, Inje University College of Medicine, Busan, Republic of Korea; 7 Department of Cancer Control and Population Health, National Cancer Center, Graduate School of Cancer Science and Policy, Goyang, Republic of Korea

**Keywords:** Microbiota, Menopause, Human papillomavirus, Microbiology, Medical research

## Abstract

In this study, we examined the difference in the vaginal microbiota of women infected with human papillomavirus (HPV), according to menopausal status. A total of 75 cervicovaginal swab samples from 38 pre- and 37 postmenopausal women with HPV infection were obtained from the Korean HPV cohort. Vaginal microbiota analysis, including microbial diversity and specific bacterial abundances, was performed using 16S rRNA gene sequencing. The mean age of the pre- and postmenopausal women were 29.5 and 55.8 years, respectively (*p* < 0.0001). *Lactobacillus* spp*.* were predominant in both groups; however, a marked decrease was observed in postmenopausal women compared to premenopausal women (44.3% vs. 74.2%). Various anaerobic bacteria also showed a relatively high abundance in the postmenopausal group; *Atopobium vagina* and *Gardnerella vaginalis* significantly increased in postmenopausal women. Interestingly, no significant differences in bacterial richness were observed between the two groups. However, significant differences in beta-diversity were observed using the Bray–Curtis (*p* = 0.001), Generalized UniFrac (*p* = 0.002), Jensen-Shannon (*p* = 0.001), and UniFrac algorithms (*p* = 0.002). Theres results indicate that postmenopausal women with HPV infection exhibited a higher degree of vaginal dysbiosis than premenopausal women. Further, HPV-infected postmenopausal women had increased vaginal microbial diversity, characterized by an increase in anaerobic bacteria and concomitant depletion of *Lactobacillus* spp.

## Introduction

The human papillomavirus (HPV) is sexually transmitted, and more than 80% of women are infected by the age of 45 years^1^. Most cases of HPV infection spontaneously resolve within 12–24 months^2^. However, HPV is a well-known risk factor for cervical cancer, and persistent HPV infection causes carcinogenesis^3^. The factors contributing to persistent HPV infection are not yet fully understood. Other epidemiological risk factors for cervical carcinogenesis include smoking, multiparity, number of sexual partners, oral contraceptive use, immunosuppression, and sexually transmitted diseases^4,5^. Among sexually transmitted infections such as *Chlamydia*, *syphilis*, *Mycoplasma genitalium*, Herpes simplex virus, and HPV infection, HPV infection is the most common^6^. While some local microbicides have been suggested for their intrinsic antiviral activity, there is no specific treatment available for the virus itself^7^.

Recent studies have shown that the vaginal microbial environment plays a role in persistent HPV infection^8–10^. A healthy vaginal microbiota is primarily composed of *Lactobacillus* spp*.* in the vaginal epithelium, thereby rendering an acidic vaginal environment owing to lactic acid production^9^. A healthy microbiota contributes to maintaining a low pH in the vagina and inhibits the growth of urogenital pathogens by preventing their colonization in the vaginal epithelium^11^. However, HPV-infected women with CIN and cervical cancer have a reduced proportion of *Lactobacillus* spp*.* and a more diverse vaginal microbiota than healthy women^12^. Evidence indicates that the likelihood of HPV persistence is higher among women with an altered vaginal microbiota. HPV infection is associated with vaginal microbiota, and cervical carcinogenesis is intricately linked to the immune response of the vaginal microbiota^13^. HPV infection may induce alterations in the microbiota of the lower genital tract through elicitation of the host mucosal immune response and initiation of genital inflammation^14^. Consequently, disruption of the vaginal environment promotes abnormal adhesion of HPV in the vagina. This sequence of events results in a local microecological imbalance that compromises the local immune function of the cervix, promoting adhesion, invasion, and colonization within an aberrant microenvironment. Subsequently, this affects the lower genital tract tissues, characterized by high vaginal pH and a non-*Lactobacillus*-dominant vaginal microbiota. This condition contributes to chronic inflammation, persistent HPV infection, and the progression of infection, potentially leading to the development of cervical cancer.

Female hormones significantly influence the vaginal microbiota, with the highest stability observed during the estrogen peak period throughout the menstrual cycle^15^. Adequate estrogen levels play a role in maintaining an intact vaginal epithelium and a healthy vaginal microbiome^16^. Estrogen plays a crucial role in preserving the collagen content of the vaginal epithelium, thereby influencing its thickness and elasticity. It also contributes to the maintenance of acidic mucopolysaccharides and hyaluronic acid, thereby maintaining moisture on the epithelial surface. When proliferating surface cells break down, glycogen is released, which serves as a substrate for *Lactobacillus*^17^. In the healthy vaginal environment of reproductive-aged women, *Lactobacillus* spp. are predominant in the vaginal microbiota. Comprehensive studies on vaginal microbiota has conclusively established that the composition of the vaginal microbiota in healthy women is dynamic and closely linked to factors such as age, race, menstrual cycle, drugs, and sexual behavior^18^.

In postmenopausal women, the vaginal environment undergoes changes due to a decrease in female hormones. This leads to a decrease in vaginal-cervical cell permeability and the occurrence of vaginal dryness^19^. The composition of vaginal microbiota also shifts in postmenopausal women. Previous studies have reported that the vaginal microbiota of healthy, postmenopausal women have a lower diversity of *Lactobacillus* spp. in their vaginal microbiota compared to premenopausal women^20,21^. Changes in the vaginal microbial environment following menopause may have a negative impact on the development of cervical cancer, especially in women with risk factors, such as HPV infection. Therefore, identifying microbiota shifts after menopause in HPV-infected women can provide helpful information for the management of such infection. To the best of our knowledge, there have been no comparative studies of the vaginal microbiota in pre- or postmenopausal women infected with HPV. In this study, we aimed to compare the microbiota composition of pre- and postmenopausal women with HPV infection using next-generation sequencing (NGS) techniques.

## Results

The sociodemographic and clinical characteristics of the study population are presented in Table 1. The mean age in the pre- group and postmenopausal groups were 29.7 and 55.7 years, respectively. The mean age at menopause was 50.6 years, and most postmenopausal women (34/37) were not receiving hormonal therapy. The mean body mass index was significantly higher in the postmenopausal group than in the premenopausal group (*p* = 0.002). Compared with the premenopausal group, the postmenopausal group had significantly higher rate of marriage and pregnancy (*p* < 0.0001). However, the premenopausal group had a significantly higher HPV vaccination rate than the postmenopausal group (*p* < 0.0001). Furthermore, the premenopausal group had a significantly higher prevalence of smoking (*p* = 0.047) and alcohol consumption (*p* = 0.003). Significant differences on vaginal intercourse (*p* = 0.025), number of sex partners (*p* = 0.025), and treatment for vaginitis in the last year (*p* = 0.01) were also observed between the groups.

**Table 1:** Sociodemographic and clinical characteristics of study population.

Category	Premenopausal group (n = 38)	Postmenopausal group (n = 37)	*p*-value
Age (year), mean ± SD	29.7 ± 5.0	55.7 ± 3.2	< 0.0001
BMI (kg/m^2^), mean ± SD	21.4 ± 2.6	23.3 ± 2.4	0.002
Marriage, n (%)			< 0.0001
Yes	13	37	
No	25	0	
History of pregnancy, n (%)			< 0.0001
Yes	10	37	
No	28	0	
HPV vaccination, n (%)			< 0.0001
Yes	25	1	
No	13	36	
Cigarette smoking, n (%)			0.047
Non-smoker	29	35	
Smoker	9	2	
Alcohol consumption, n (%)			0.003
Non-drinker	2	12	
Drinker	36	25	
Vaginal coitus for previous 1 year, n (%)			0.025
Yes	35	28	
No	2	10	
Sex partner for previous 1 year, n (%)			0.025
1	28	35	
≥ 2	10	2	
Vaginitis for previous 1 year, n (%)			0.001
Yes	10	0	
No	28	37	

SD, standard deviation; BMI, body mass index; HPV, human papilloma virus.

The vaginal microbiota composition at the genus level between the two groups were compared (Table 2). *Lactobacillus* was the most abundant taxa in both groups. However, the proportion of *Lactobacillus* in the postmenopausal group was significantly lower than that in the premenopausal group (44.3% vs. 74.2%). The abundance of *Gardnerella* (15.7% vs. 7.3%) and *Atopobium* (11.3% vs. 1.5%) were significantly higher in the postmenopausal group than in the premenopausal group. *Bifidobacterium* was only observed in the premenopausal group, whereas *Escherichia*, *Sneathia*, *Megasphaera*, *Dialister*, *Aerococcus*, and *Phyllobacterium* were observed in the postmenopausal group.

**Table 2:** Comparison of taxonomic composition between pre- and post-menopausal women with HPV infection.

Genus	Premenopausal group (%)	Postmenopausal group (%)
*Lactobacillus*	74.2	44.3
*Gardnerella*	7.3	15.7
*Bifidobacterium*	3.7	–
*Streptococcus*	3.4	1.2
*Prevotella*	3.4	6.6
*Atopobium*	1.5	11.3
*Escherichia*	–	5.3
*Sneathia*	–	5.0
*Megasphaera*	–	2.0
*Dialister*	–	1.2
*Aerococcus*	–	1.2
*Phyllobacterium*	–	1.1
Others (under 1% in average)	6.5	5.1

HPV, human papilloma virus.

**Figure 1:**
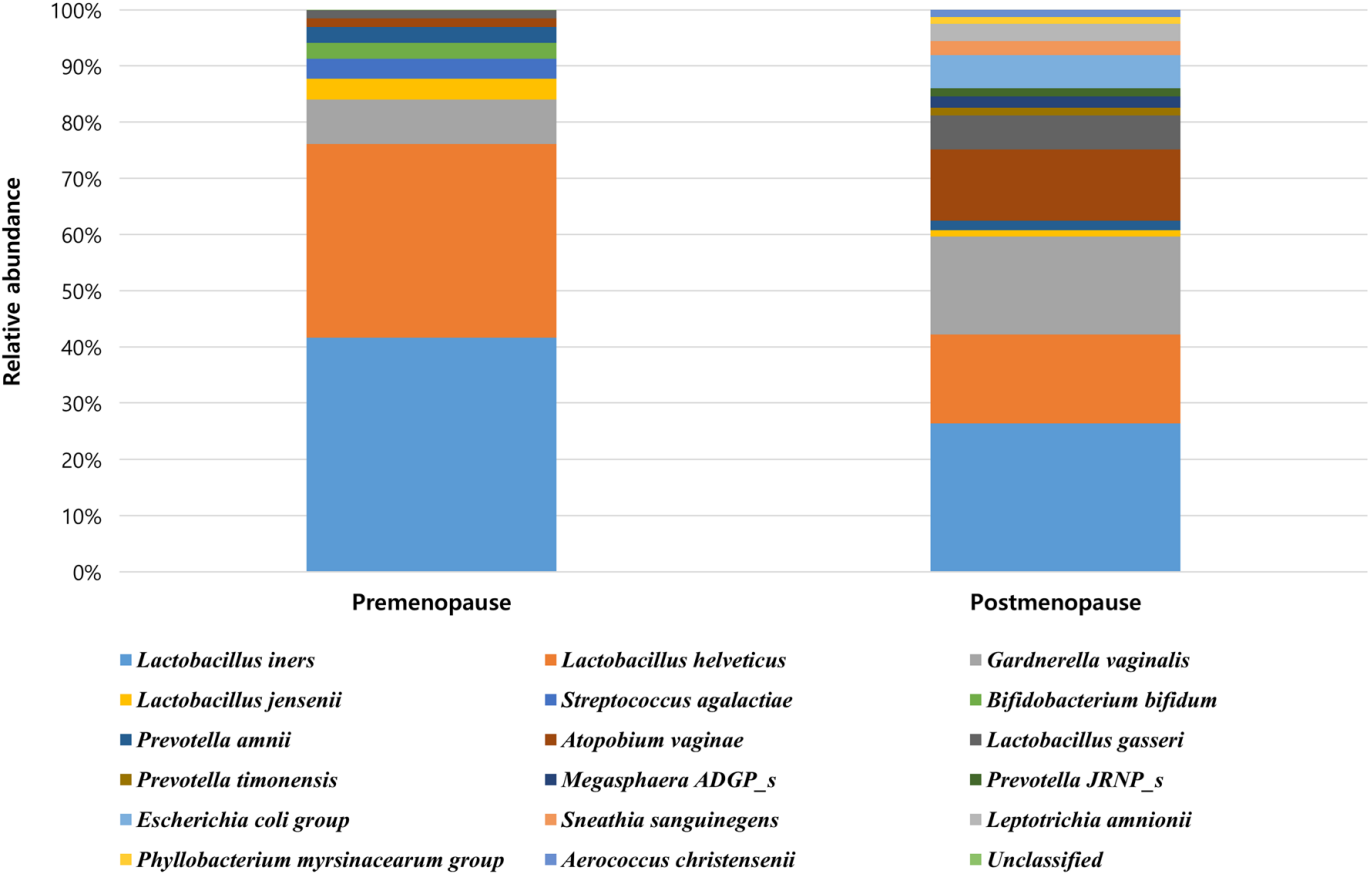
The proportion of operational taxonomic units of the vaginal microbiota in pre- and postmenopausal women with HPV infection. HPV, human papillomavirus.

The relative proportions of common microbial taxa at the species level in each group are shown in Fig. 1. In the premenopausal group, *Lactobacillus iners* (41.6%) and *Lactobacillus helveticus* (31.4%) were the most abundant, whereas a few other bacteria were observed in small proportions. Microbial diversity was higher in the postmenopausal group than in the premenopausal group. In the postmenopausal group, the abundance of *Lactobacillus iners* (26.3%) and *Lactobacillus helveticus* (14.1%) decreased, whereas anaerobic bacteria such as *Gardnerella vaginalis* (15.7%), *Atopobium vaginae* (11.3%), *Escherichia coli* (5.3%), *Leptotrichia amnionii* (2.7%), and *Sneathia sanguinegens* (2.3%) were observed at a relatively high abundance.

**Figure 2:**
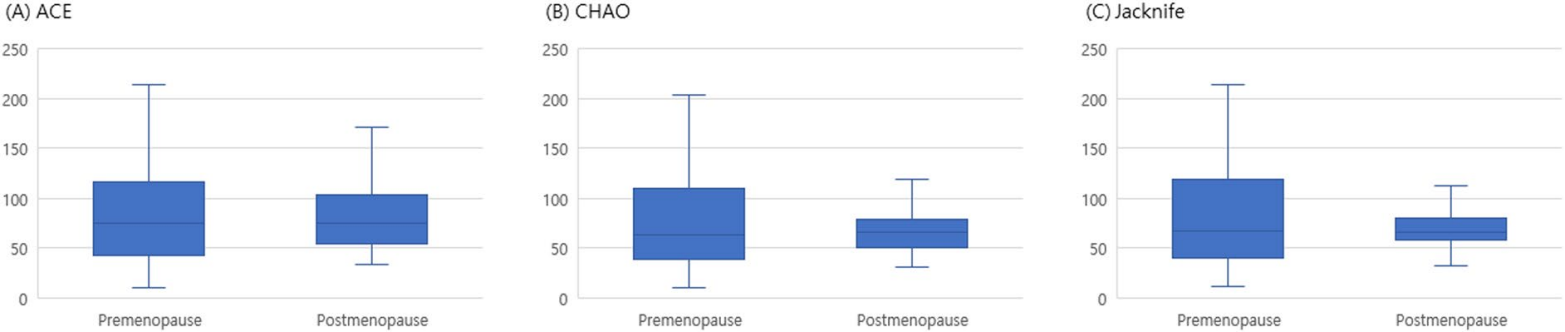
Alpha-diversity of microbial communities in each group. The Shannon diversity index according to the groups. (**A**) The mean Shannon diversity index was compared between HPV negative and HPV positive groups. (**B**) Shannon diversity index was calculated according to the results of histopathological diagnosis.

**Figure 3:**
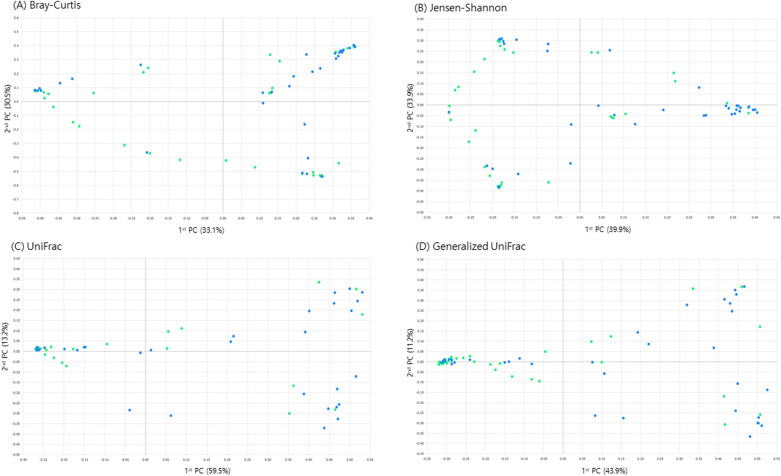
Beta-diversity of microbial communities in HPV-infected women. Principal coordinate analysis (PCoA) of the bacterial communities are displayed in a 2D diagram. (**A**) Bray–Curtis, (**B**) Jensen-Shannon divergence, (**C**) UniFrac, and (**D**) Generalized UniFrac. The green and blue color indicate pre- and postmenopausal women, respectively.

Figure 2 shows the alpha-diversity of each group. Bacterial richness was not different between the two groups. However, significant differences were found between the pre-menopausal and post-menopausal groups using principal coordinate analysis (PCoA) plots with beta-diversity analyses (Fig. 3). The study employed several algorithms to assess beta-diversity, including Bray–Curtis, Generalized UniFrac, Jensen-Shannon, and UniFrac and the significance of beta-diversity was calculated by Permutational Multivariate Analysis of Variance (PERMANOVA). The statistical significance of the differences observed using these algorithms is demonstrated by the Bray–Curtis (*p* = 0.001), Generalized UniFrac (*p* = 0.002), Jensen-Shannon (*p* = 0.001), and UniFrac algorithms (*p* = 0.002). The results indicated that the distribution of vaginal microbiota was significantly different between pre- and postmenopausal women with HPV infection.

## Discussion

Microbiota composition and alterations in pre- and postmenopausal women infected with HPV remain unclear. The results of this study showed that postmenopausal women with HPV infection exhibited vaginal dysbiosis compared with premenopausal women with HPV infection. Although *Lactobacillus* was most abundant in both groups, postmenopausal women exhibited a lower abundance of this taxon and a more diverse composition. Notably, postmenopausal women had an increased vaginal microbial diversity, characterized by an increase in the abundance of anaerobic bacteria and concomitant depletion of *Lactobacillus* spp.

With the onset of menopause and decreased estrogen levels, the vaginal epithelium undergoes thinning. The reduced number of epithelial cells leads to decreased exfoliation in the vagina, causing an increase in pH. This shift results in a decline in the abundance of *Lactobacillus* and overgrowth of other bacteria, including *Prevotella*, unclassified *Lactobacillaceae*, *Escherichia*, *Pseudomonas*, *Proteus*, *Finegoldia*, and *Atopobium*^22^. In the present study, we observed a reduced abundance of lactobacilli, accompanied by a relatively higher abundance of various anaerobic bacteria, such as *Gardnerella vaginalis*, *Atopobium vaginae*, *Escherichia coli*, *Leptotrichia amnionii*, and *Sneathia sanguinegens* in the postmenopausal group compared to the premenopausal group. Several studies have reported an association between elevated vaginal microbiota diversity and the prevalence of HPV infection and/or cervical abnormalities. An increased abundance of *Lactobacillus crispatus* has been demonstrated to correlate with a lower prevalence of HPV^23^. The prevalence of widespread HPV infection was associated with reduced abundance of *Lactobacillus* spp., with a notable increase in anaerobes belonging to the genera *Prevotella* and *Leptotrichia*^24^. Our previous study on vaginal microbiota revealed that the vaginal microbial composition in women with cervical intraepithelial neoplasia (CIN) or cervical cancer showed elevated microbial diversity^12^. Compared to the normal group, patients with cervical cancer and CIN with HPV infection had a marked decline in *Lactobacillus crispatus* and increased *Atopobium vaginalis*, *Dialister invisus*, *Finegoldia magna*, *Gardnerella vaginalis*, *Prevotella bucalis*, and *Prevotella timonensis*. Another study on the vaginal microbiota showed that with the progression of cervical dysplasia, vaginal microbiota environment alterations occur^25^. Specifically, in women with high-grade CIN and cervical cancer, *Lactobacillus* was notably reduced, whereas other bacteria, including anaerobic bacteria, such as *Prevotella* and *Megasphaera,* significantly increased.

In this study, postmenopausal women exhibited increased vaginal microbial diversity, marked by a rise in anaerobic bacteria and subsequent depletion of *Lactobacillus* spp., in comparison to premenopausal women. Alpha-diversity was similar between pre- and postmenopausal women with HPV infection, suggesting comparability in the intra-diversity or microbial richness. Specifically, the number and abundance of microbial species within individual samples did not differ significantly between groups. However, the beta-diversity was higher in postmenopausal women with HPV infection, indicating a more substantial variation in microbial composition among groups. This suggests a greater degree of variability or heterogeneity in the microbial profiles of postmenopausal women with HPV infection. Considering the common factors of HPV infection, changes in the vaginal microflora seemed to escalate with menopause in this study. Menopause can be considered a factor that significantly alters microbial distribution in the vaginal environment of HPV-infected women.

The influence of menopause on the vaginal environment is presented not only by hormonal changes but also by various epidemiological factors distinct from those affecting premenopausal women. Within our study population, disparities were observed in epidemiological factors, including smoking, drinking habits, sexual behavior, and treatment of vaginitis between pre- and postmenopausal women. In particular, bacterial vaginosis is known to be significantly associated with sexual behavior, and decreasing the frequency of unprotected sexual encounters may lower the incidence and recurrence of bacterial vaginosis^26^. Despite the prevalence of epidemiological factors that adversely impact the vaginal ecosystem being higher in the premenopausal group, this study revealed a higher vaginal dysbiosis in postmenopausal women with HPV infection. This suggests that menopause significantly influences the balance of vaginal microflora in HPV-infected women, surpassing the impact of other epidemiological factors. Persistent HPV infections are common among postmenopausal women^27^. A previous study indicated that menopausal status is an independent predictor of HPV persistence following the surgical treatment of high-grade CIN^28^. Further, the duration of HPV persistence may be closely linked to cellular immune functions^29^. Insufficient immunological control of HPV infection, resulting in viral persistence, is likely to be a crucial factor influencing the risk of progression to cervical neoplastic disease.

To the best of our knowledge, no studies have reported an association between menopause and the vaginal microbiota in women infected with HPV. Overall, this study identified a disparity in the vaginal microbiota between pre- and postmenopausal women with HPV infection. Although premenopausal women have more epidemiological factors that adversely affect their vaginal microbial environment, more changes in the vaginal microbial environment have been observed in postmenopausal women. Postmenopausal women with HPV infections appear to be more susceptible to dysbiosis of the vaginal microbiota. This susceptibility is attributed not only to the adverse effects of HPV infection but also to alterations in the vaginal environment resulting from a decline in female hormones. Consequently, HPV-infected postmenopausal women may have an increased risk of developing CIN and cervical cancer, which is consistent with the heightened incidence of cervical cancer in elderly women. Therefore, the potential risk of cervical carcinogenesis should be carefully managed in women with persistent HPV infection, even after menopause. Balancing the vaginal microbiota in HPV-infected postmenopausal women may be important for preventing HPV-related diseases. Further research in this area is necessary.

## Methods

### Specimen collection

A total of 75 cervicovaginal swab samples were collected from 38 pre- and 37 postmenopausal women with HPV infection from a Korean HPV cohort^30^. This cohort included HPV-infected women aged 20–60 years with low-grade abnormal cervical cytology results (atypical squamous cells of undetermined significance, ASC-US and low-grade squamous intraepithelial lesion, LSIL). Participants were interviewed by a trained examiner and completed a questionnaire. Women with gynecological cancer, pregnancy, chronic diseases, drug dependency, psychological problems, or insufficient questionnaire data were excluded. Patients who are currently on antibiotics or have recently taken antibiotics were excluded. All study participants provided an informed consent, and the study protocol was approved by the Institutional Review Board (No. KUMC 2021-08-038).

### DNA extraction and NGS

Genomic DNA (gDNA) was extracted from the cervicovaginal swabs using the MagNA Pure 96 DNA and Viral NA Small Volume Kit (Roche Applied Science, Mannheim, Germany), according to the manufacturer’s instructions, and stored at − 80 °C until further analysis. DNA concentration and purity were analyzed using a Qubit™ dsDNA HS Assay Kit (Thermo Fisher Scientific, Waltham, MA, USA) and Nanodrop (Thermo Fisher Scientific, Waltham, MA, USA), respectively.

Library preparation was performed according to the Illumina 16S Metagenomic Sequencing Library protocol. The gDNA was amplified using primers specific for the V3–V4 region of the 16S rRNA, and a subsequent limited-cycle amplification step was performed for the added multiplexing indices and Illumina sequencing adapters. The primer sequences were as follows: Forward primer, 5ʹ-TCGTCGGCAGCGTCAGATGTGTATAAGAGACAGCCTACGGGNGGCWGCAG-3ʹ; reverse primer, 5ʹ-GTCTCGTGGGCTCGGAGATGTGTATAAGAGACAGGACTACHVGG.

GTATCTAATCC-3ʹ. PCR products were then normalized and pooled using the Qubit™ dsDNA BR Assay Kit (Thermo Fisher Scientific, Waltham, MA, USA), and library sizes were verified using the TapeStation D1000 DNA ScreenTape (Agilent, USA). The amplified genes were sequenced using the MiSeq DX platform (Illumina, San Diego, CA, USA). Paired-end sequencing was performed using MiSeq Reagent kit V3 (2 × 300 bp/600 cycle) (Illumina, San Diego, CA, USA).

### Bioinformatic analysis

NGS produced a total of 7,297,312 paired-end reads. Taxonomic profiling was performed for each sample, the intra- and inter-group diversities were calculated, and the differentially represented taxa were determined using EzBioCloud. Operational taxonomic units (OTUs) at 97% sequence identity were assigned using EzBioCloud public database. Alpha-diversity and species richness were evaluated using Shannon’s index, while beta-diversity was calculated using Bray–Curtis, Generalized Unifrac, Jensen-Shannon, and Unifrac.

### Statistical analyses

Statistical analysis was performed using SPSS for Windows (version 17.0; SPSS Inc., Chicago, IL, USA). Categorical variables are presented as numbers and percentages, while continuous variables are presented as mean and standard deviation (SD). The *t*-test and chi-square test were used to analyze continuous and categorical variables, respectively. *p* < 0.05 was considered statistically significant.

### Ethical approval

The study was approved by the Institutional Review Board of the Konkuk University Medical Center (No. KUMC 2021-08-038). All procedures performed in this study were in accordance with the ethical standards of the institution and with the 1964 Helsinki declaration and its later amendments. Informed consent was obtained for all participants.

## Data Availability

The datasets use and/or analysed during the current study are provided by the corresponding author on a reasonable request.
